# Neurolinguistic and acoustic analysis of articulatory impairments in Arabic speech disorders

**DOI:** 10.3389/fnhum.2025.1638363

**Published:** 2025-10-07

**Authors:** Jeehaan Algaraady, Mohammad Mahyoob, Mohammad Zubair Khan

**Affiliations:** ^1^Languages and Translation Department, Taiz University, Taiz, Yemen; ^2^Languages and Translation Department, Taibah University, Madina, Saudi Arabia; ^3^Department of Computer Science, Applied College, Taibah University, Medina, Saudi Arabia; ^4^King Salman Centre for Disability Research, Riyadh, Saudi Arabia

**Keywords:** neurolinguistic analysis, acoustic deviations, speech disorders, pharyngeal sounds, Arabic phonetics

## Abstract

**Background:**

Pharyngeal sounds, integral to Arabic phonetics, require precise vocal tract coordination, posing significant challenges for individuals with speech disorders.

**Objective:**

This study investigates the neurolinguistic and acoustic characteristics of pharyngeal sound production in Arabic speakers with speech impairments, aiming to elucidate the impact of neurological disruptions on articulatory precision.

**Methods:**

A comparative study was conducted with 20 participants (10 with speech disorders, 10 with typical speech). Acoustic analysis, including spectrographic evaluation, was used to quantify deviations in pharyngeal sound production. Concurrently, neurolinguistic assessments, which included neurological evaluations, identified disruptions in neural pathways governing speech motor control.

**Results:**

Individuals with speech disorders exhibited significant neuromotor deficits, correlating with distinct acoustic deviations in pharyngeal sound production. These findings highlight the synergy of neurolinguistic and acoustic approaches in identifying underlying mechanisms of speech impairment.

**Conclusion:**

By integrating neurolinguistic and acoustic analyses, this study establishes a novel framework for diagnosing and treating pharyngeal sound disorders in Arabic speakers. The results inform targeted therapeutic interventions and the development of assistive technologies, advancing clinical outcomes in speech-language pathology.

## 1 Introduction

The interaction of neurolinguistic and acoustic analysis in the study of speech disorders bridges the gap between brain function and the physical production of speech. Neurolinguistics focuses on understanding how brain structures and neural pathways control speech, offering an understanding of disruptions in individuals with disorders (Friederici, 2011). These disruptions often affect the motor control required for speech articulation, where acoustic analysis quantitatively assesses speech output through measurements like frequency, intensity, and formant structures (Kent, 2000). By integrating these two analyses, researchers can connect specific neural deficits to the corresponding acoustic anomalies, offering a multidimensional view of speech disorders. This combined approach allows for more accurate diagnostic frameworks and the development of targeted therapeutic interventions. For instance, identifying the acoustic signature of a disordered sound and its neurolinguistic basis enables the creation of individualized speech therapy protocols, which can address both the neural and acoustic aspects of impaired speech (Fougeron et al., 2022; Youn et al., 2021). This integration holds significant potential for advancing the treatment of speech impairments and refining our understanding of speech production mechanisms.

Individuals with speech disorders often face challenges stemming from underlying brain problems that disrupt the normal processes of speech production and language comprehension. These disorders can arise from various neurological conditions, such as stroke, traumatic brain injury, developmental disorders, or neurodegenerative diseases, all of which affect regions of the brain responsible for speech-motor control, language processing, and auditory feedback (Duffy, 2012). For example, damage to Broca’s area in the frontal lobe can impair the motor planning required for articulation, leading to conditions like apraxia of speech, where individuals struggle to produce coordinated speech sounds despite understanding language (Ziegler, 2002). Similarly, issues in the basal ganglia or cerebellum can cause dysarthria, where muscle weakness or coordination problems result in slurred or slow speech (Diepeveen et al., 2022; Chilosi et al., 2022; Maas et al., 2008). These brain-related disruptions highlight the importance of understanding the neurological basis of speech disorders, as therapeutic interventions often require a multidisciplinary approach to address both the cognitive and motor aspects of speech impairment.

In Arabic phonetic structure, pharyngeal sounds are distinctive sounds that demand sophisticated control over specific vocal tract mechanisms. These sounds complicate the ability of individuals with speech disorders to produce Arabic phonetics accurately. Arabic is among the most prevailing Semitic languages, distinguished by its distinctive phonetic inventory and sophisticated syntactic and morphological structure (Watson, 2007). Notably, its distinct phonetic features, such as pharyngeal, emphatic, and uvular consonants, and short and long vowels, need to be revised to analyze and process Arabic speech (Selouani and Caelen, 1998). The complicated nature of Arabic phonetics and phonology introduces a fascinating field for exploring speech disorders and their effect on an individual’s communication skills. In the Arabic language, pharyngeal sounds are particularly notable, and they are rare in the phonetic inventories of most languages, making Arabic especially significant for phonetics and speech pathology studies.

For speakers of Arabic, the production of pharyngeal sounds poses specific challenges due to the complex physiological demands involved, making the availability of speech carriers to pronounce these sounds accurately a particular problem (Versteegh, 2014). Arabic lung sounds originate from the soft palate, requiring precise neuromuscular control. Any deviation from this equilibrium, whether innate or later acquired, can lead to specific speech acts. The precise phonemes of such abnormalities are still unknown, and the limited research devoted to these particular phonemes in speech analogy is further controversial (Stone, 1997). Troubles in generating these sounds can markedly affect people’s communication. Introducing immediate solutions for individuals with speech disorders is vital as soon as impairment is detected. Generally, many causes, underpinnings, or processes underlying speech disorders, including delays in speech development, neurological issues, or anatomical differences, lead to significant challenges in personal, academic, and social settings (McCormack et al., 2009).

Moreover, speech disorders can potentially lead to other social anxiety disorders and avoidance behavior (Dochert et al., 2013). The existing analyses focused on Arabic speech disorders in general, either investing in machine learning methods or not, are limited; for example (Amayreh and Saleh, 2014; Terbeh et al., 2016; Hammami et al., 2020; Alqudah et al., 2020; Shareef and Al-Irhayim, 2022). However, the Arabic language is one of the fifth most widely used languages (Al-Zabibi, 1990). This study investigates the difficulties of producing pharyngeal sounds, their characteristics, and the articulatory and acoustic features attributed to these characteristics in Arabic speakers with speech disorders.

The production of pharyngeal sounds, which requires precise control of the vocal tract muscles, is closely tied to the functioning of the nervous system, particularly the brain’s motor pathways. Pharyngeal sounds demand coordinated activity between the brain’s motor cortex, brainstem, and cranial nerves to regulate tongue and pharynx movement. Any disruption in these neural pathways, from neurological damage or developmental issues, can lead to physical difficulties in producing these complex sounds. For example, individuals with neurological impairments may struggle with the fine motor control needed for accurate pharyngeal sound production, leading to the distorted or incomplete articulation of these sounds (Kent, 2000). Analyzing pharyngeal sounds in speech production and their acoustic results in affected individuals highlights the intricate relationship between neural control and the physical articulation of speech. The authors attempt to thoroughly explore the interaction between speech disorders and Arabic’s pharyngeal phonetics to advance academic insight and practical clinical approaches.

The rest of the study is organized as follows: Section 2 introduces the related work. Section 3 details the methodology used for this acoustic analysis and evaluation. Section 4 presents the results and discussion. Section 5 concludes this investigation and outlines directions for future research.

### 1.1 Guttural sounds in Arabic

In Arabic, Guttural sounds include a group of consonants produced in the back of the vocal tract (McCarthy, 1991). These sounds comprise pharyngeal, velar, uvular, and glottal sounds. The peculiarity of this group in Arabic lies in the specificity of their place of articulation, which gives them a “deep” or “throaty” quality. Our investigation focuses on the pharyngeal sounds, as illustrated in Table 1.

**Table 1:** Distinctive features of pharyngeal and velar.

Sound in Arabic	IPA symbol of the sound	Place of Articulation	Manner of Articulation	Voicing	Consonantal	Sonorant	Continuant	Velar/Dorsal	Pharyngeal
ح (Ḥā’)	ħ	Pharyngeal	Fricative	Voiceless	Yes	No	Yes	No	Yes
ع (‘Ayn)	ʕ	Pharyngeal	Fricative	Voiced	Yes	Yes	Yes	No	Yes

### 1.2 Phonetic and phonological distinctive features

Distinctive features are specific phonological and phonetic properties that distinguish one phoneme from another within a particular language (Roach, 2009). These features allow us to classify and describe how sounds are produced and perceived. Moreover, they help analyze and understand the contracts between sounds, their function, and their contribution to the language’s meaning and structure. These distinctive features define the sounds and influence how they interact within words and sentences, affecting aspects like stress, rhythm, and intonation in Arabic. Table 1 below presents an overview of the distinctive features of guttural sounds in Arabic, focusing on pharyngeal sounds (/ħ/, / ح/ and / ʕ/, / ع/).

## 2 Aim of the study

This exploratory study aims to investigate the acoustic and neurolinguistic challenges individuals with speech disorders face when articulating pharyngeal sounds in Arabic, focusing on Saudi Arabic, and assess their impact on speech clarity and communication. By integrating acoustic analysis with neurolinguistic insights, the study seeks to deepen our understanding of the neurological and motor control complexities underlying speech production in Arabic speakers with speech impairments, contrasting typical and disordered speech patterns. The rich acoustic and neurolinguistic data generated will be the foundation for developing machine-learning models capable of classifying, predicting, diagnosing, and monitoring treatment progress for Arabic pharyngeal sound disorders. The study aims to advance innovative diagnostic and therapeutic solutions for clinicians and researchers working with speech disorders by integrating machine learning methodologies and speech pathology.

## 3 Related work

The Arabic speech disorders research has mainly focused on prevalence, diagnostic techniques, public awareness, and technological approaches for early detection and intervention. However, literature in this area is scarce, especially concerning comprehensive frameworks integrating phonetic, acoustic, and computational methodologies in Arabic contexts. The preliminary research, including Amayreh and Saleh (2014), gave a broad overview of speech disorders in Arabic speakers, including prevalence and awareness. Their results showed there was a relatively high prevalence (7.5%) of students with speech impairments in Jordan, the largest group being those with voice and articulation disorders. This study offered important epidemiological information but did not go into acoustic or articulation details.

Subsequently, computational methods reached prominence. Terbeh et al. (2016) proposed a probabilistic-phonetic model with emphasis placed on the acoustic features to distinguish between native Arabic speakers with voice pathologies and non-native learners. Their model detected pronunciation disorders with an accuracy of 95%, confirming the efficiency of probabilistic acoustic modeling. Similarly, Selouani and Caelen (1998) proposed a high-level speech recognition system specially designed for Arabic-speaking children with speech disorders. They achieved a staggering accuracy of 97.99% by using Mel frequency cepstral coefficients (MFCCs) and LSTM networks. Due to this, it became convincing that machine learning promises to detect speech impairments robustly; however, such studies mainly dealt with isolated speech error types instead of complete phonological systems.

An extension of the computational paradigms focusing nowhere else but on those error nuances in the articulation of the Arabic phoneme /r/has been made by Hammami et al. (2015, 2020) and Abdo et al. (2023). To specify sound errors across different word positions, they emphasized accurate acoustic analyses that consider position specificity, given the sound classification approach that utilized MFCCs, probabilistic classifiers, and ensemble techniques such as bagged tree classifiers. Most notably, the studies revealed problematic inconsistencies in Arabic phonetic articulation; on the other hand, they focused on one phoneme only, thereby neglecting broader phonological considerations.

In preparation for complementary efforts, Alqudah et al. (2020) have established the Phonetic Dictionary Generator (PDG), which filled in the methodological gap of producing a fine resource for automatic speech recognition systems for both normal and disordered Arabic speech. This development highlighted the need for lexical bases capable of handling the variability of Arabic phonetically.

The studies highlight several key points for consideration: the positive impact of computational models on speech disorder diagnosis, the need to allocate specific databases and resources that account for Arabic phonetic peculiarities, and the importance of early intervention supported by precise diagnostic tools. Significant gaps remain, particularly in the form of more general neurolinguistics and acoustic-phonetic analyses that consider several Arabic guttural sounds or account for more general articulatory dynamics in speech disorders, which have been overlooked. And so far, very few studies integrate diagnostic accuracy with therapy explicitly designed for Arabic phonology.

Considering these gaps, the study attempted to make a comprehensive acoustic analysis of the Arabic guttural (**/ħ/ and** /ʕ/) to contribute to subtle insights into articulatory precision and acoustic variability among speech-impaired individuals. The results here serve to widen the present framework and interrelate epidemiologic, neuro-acoustic, computational, and therapeutic approaches to work toward better diagnostic and intervention tools in Arabic speech disorders.

## 4 Materials and methodology

### 4.1 Participants

The study comprised 20 native Saudi Arabic-speaking participants, equally divided by speech disorders (n = 10) and control groups (n = 10), aged 6–37. Participants’ inclusion criteria indicated that all participants had to be native Saudi Arabic speakers able to follow verbal instructions. The diagnosis included articulation disorders, sound distortion, and dysarthria, which indicated a moderate to severe level of articulation disorders, phonological disorders, anarthria, and dysarthria, mainly targeting pharyngeal consonants. The control group hosted candidates with no history of speech, language, hearing, or other deficits. Informed consent was sought from all participants or their guardians before the commencement of the study. The Taibah University Research Ethics Committee approved the protocol. Inclusion criteria indicated that all participants had to be native Saudi Arabic speakers able to follow verbal instructions. Participants in the speech disorder group were clinically diagnosed with articulation disorders, phonological disorders, anarthria, and dysarthria (from moderate to severe level), primarily affecting pharyngeal consonants by a certified speech-language pathologist (SLP). Exclusion criteria ruled out those with comorbidities that could influence the analytical aspect of speech, such as autism spectrum disorder, hearing impairment or loss, or severe developmental delays.

Table 2 below summarizes the participants’ demographic and clinical characteristics. The speech disorder group was matched with the control group for age and gender to minimize confounding variables.

**Table 2:** Summarizes participant demographics and clinical characteristics.

Group	*N*	Age range (Years)	Mean Age (SD)	Gender (M/F)	Diagnosis	Severity (Moderate/Severe)
Speech disorder	10	6–37	18.2 (9.5)	4 M/6F	Articulation disorders Phonological disorders Anarthria Dysarthria	6 Moderate, 4 Severe
Control	10	6–37	18.4 (9.7)	4 M/6F	None	N/A

### 4.2 Study design

The study implemented a parallel-group design where participants were assigned to either the speech disorder or the control group based on clinical diagnosis, using a 1:1 allocation ratio. Investigators who handled the acoustic data were blinded to group assignments. The choice of sample size (n = 20) was based on feasibility and consistency with previous research.

Voice samples were recorded in an acoustically controlled environment to retain the quality of the recordings. The sessions took place in a soundproof booth lined with acoustic foam panels to inhibit echo and ambient noise. A directional microphone, fitted with a pop filter to shield plosive sounds and background interference, was used. Protection against air blasts and distortion was afforded by placing the microphone at 15–30 cm from the mouth of a participant at an angle of about 30 to 45 degrees. Each participant or recording session lasted 10–20 min with the presentation of standardized stimuli ranging from isolated sounds, words, and sentences to spontaneous speech, keeping the stimuli consistent for every participant.

*Recording conditions:*

- Sampling frequency: 44,100 Hz.
- Bit depth: 16 bits per sample.
- File format: .wav.
- Equipment: laptops with SSD storage, high-fidelity sound cards, and High-quality microphones.

### 4.3 Acoustic analysis

- The acoustic data were analyzed with Praat (version 6.3.17), a tool widely used in phonetic and phonological research. Each voice recording was segmented and labeled manually to guarantee a precise acoustic analysis. The following characteristics were obtained and measured:
- Fundamental measures: Pitch, intensity, formant frequencies (F1, F2, F3).
- Variability and quality measures: Jitter (local), shimmer (local), HNR (harmonics-to-noise ratio). These measures allowed for a detailed comparative analysis of how the pharyngeal sounds are produced by the two groups, with an emphasis on the variability, clarity, and stability of the speech signals. All analyses were carried out blind to the participants, aiming at reducing the risk of observer bias. It is admitted that the findings’ limited generalizability stems from the small sample size. However, this study was aimed at contributing to the literature as a baseline study; therefore, future work will attempt to increase the participant numbers, encompass a wider range of speech disorders and severities, and carry out cross-dialectal studies. Longitudinal studies are planned to observe speech development and therapeutic efficacy over time.

## 5 Analysis and discussion

In this study, acoustic analyses were performed automatically utilizing Praat, a software tool widely used by phoneticians and linguists for studying the acoustic properties of speech and facilitating the study of speech physical indices and perceptual cues (Boersma and Weenink, 2023). This software provides complicated numerical and visual explorations of the audio recordings via waveforms and spectrograms. Acoustic analysis can be informative because it affords quantitative analyses that carry the potential for subsystem description and for determining the correlations of perceptual judgments of intelligibility, quality, and speech disorder type (Kent et al., 1999).

Quantitative data from the acoustic analysis is statistically analyzed using statistical software packages to identify significant differences between healthy and disordered speech. Descriptive statistics summarize the acoustic features of the selected sounds, and inferential statistics compare the speech disorder group with the control group. Qualitative observations from articulatory analysis complement the acoustic data, providing insight into the physical production of these sounds. The waveform illustrates sound pressure levels (loudness) and shows changes in amplitude over time, indicating increased speech loudness. Meanwhile, intensity interval indicates increased vocal effort or stressed syllables. Meanwhile, the spectrogram provides a time-varying representation of the frequency spectrum, indicating the presence and intensity of frequencies. In addition, basic acoustic features, such as solid roundness, pitch, and other key parameters, are covered below.

### 5.1 An acoustic analysis of the sound (/ħ/, / ح/)

**Figure 1:**
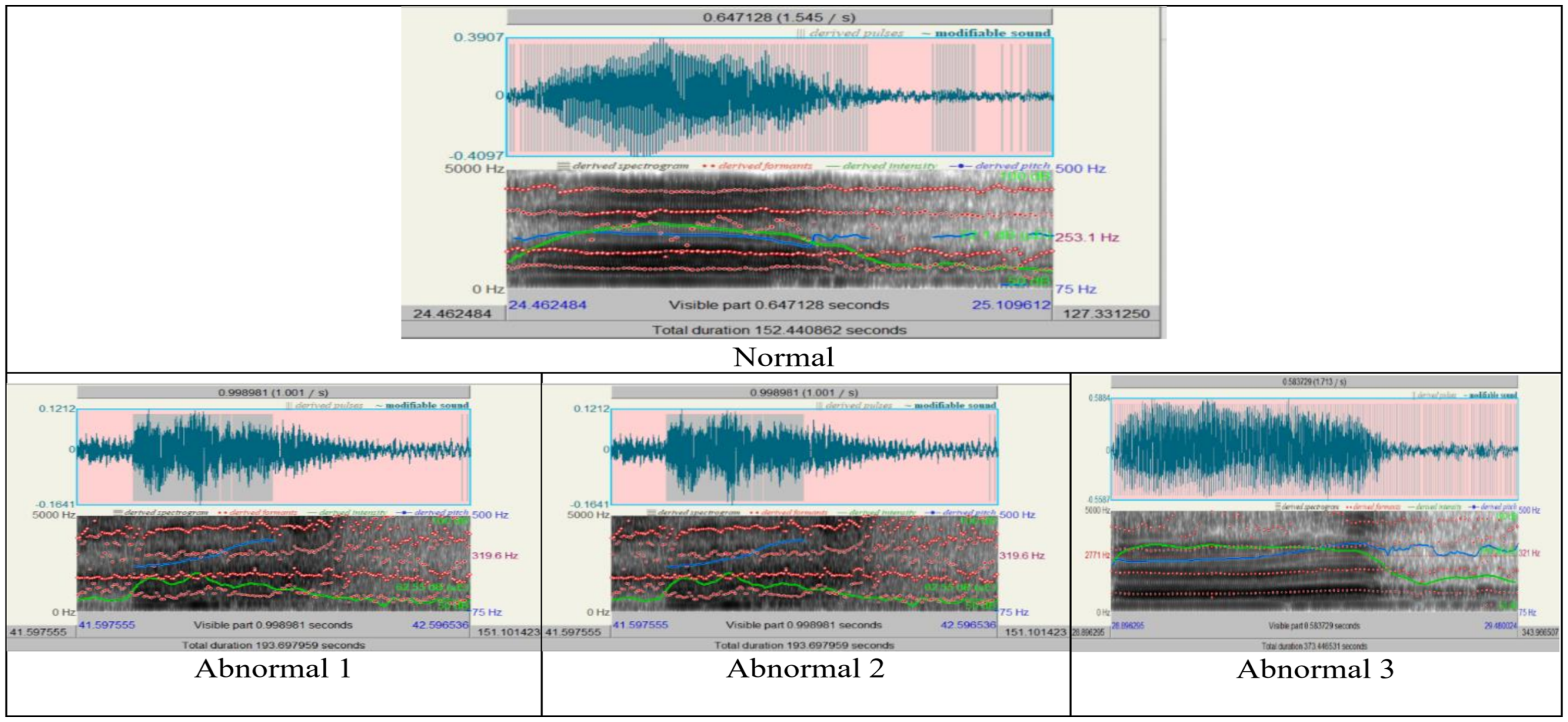
The acoustic analysis of speech sound / ħ /, “/ ح /”.

An acoustic study was performed on the Arabic pharyngeal sound (/ħ/) to distinguish between normal and disordered speech productions. The spectrographic data shown in Figure 1 represents one normal speaker and three abnormal speakers. Each panel consists of a waveform, spectrogram, pitch tracking, and formant frequencies. While the waveform shows variations in amplitude with changes in speech intensity, the spectrogram displays how frequency varies with time; pitch tracking shows how steady or variable the pitch is, and the bright bands on the spectrogram represent the formants. Normal speech exhibits a stable pitch track, a clear waveform, and well-defined formants. Any abnormalities cause disruptions to pitch stability, amplitude variations, and even a complete interruption in voicing, which is observed as fragmented pitch lines and irregularities in the spectrogram.

**Figure 2:**
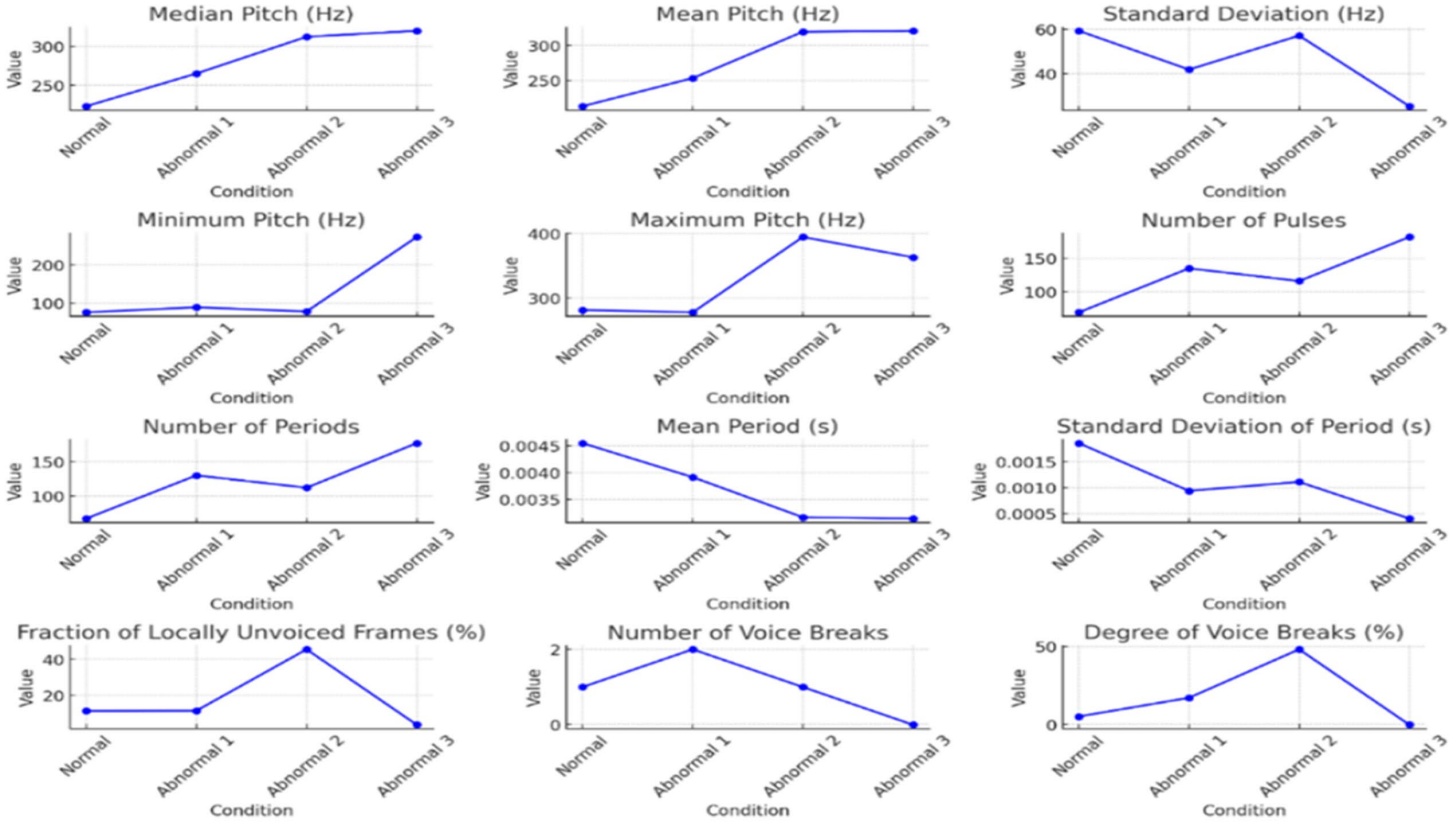
The Pitch, pulse, and voicing parameter variations across different conditions.

**Figure 3:**
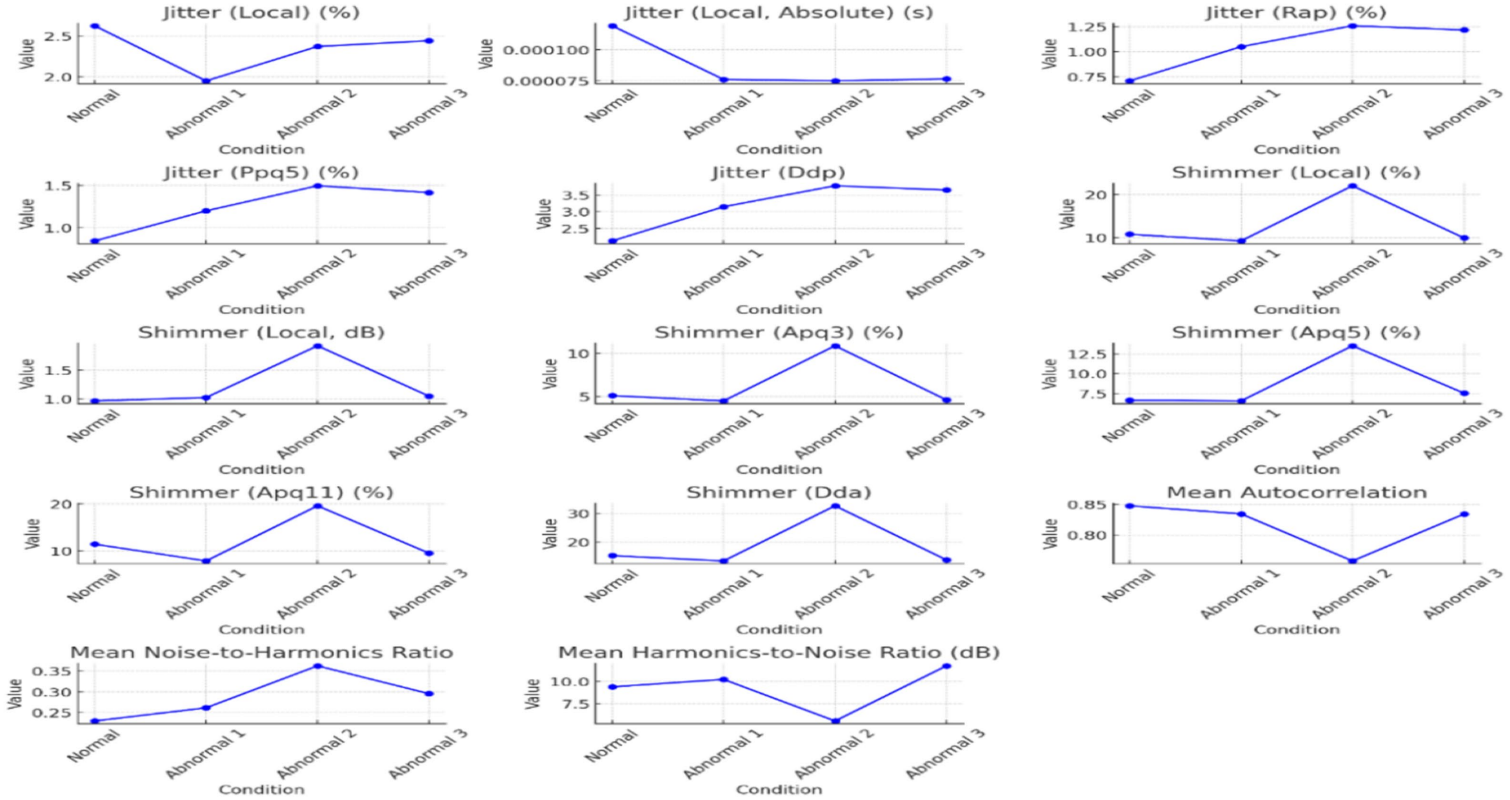
The jitter, shimmer, and harmonicity parameters across the conditions of the sounds / ħ /.

Quantitative measurement of acoustic parameters further emphasized these differences, as shown in Tables 3, 4 and represented in Figures 2, 3 below. From normal to abnormal conditions, exceptionally high in Abnormal 3, the values of pitch parameters (median, mean, minimum, maximum, and standard deviation) showed an upward trend in the abnormal conditions. Abnormal cases exhibiting high values of pitch signify vocally thicker folds or tension in vocal fold function; abnormal speakers lose control of low-pitch tones and in strained vocal production. The number of pulses and periods, which represent vocal fold vibration, showed a significant increase in Abnormal 3, probably due to compensatory adjustments in response to changes induced by pathology in the voice. A decrease in the mean period from normal to abnormal indicated that the vocal fold vibrations were faster and stiffer in the abnormal status.

**Table 3:** The pitch, pulse, and voicing across the conditions of the sound / ħ /.

Acoustic features	Parameter	Normal	Abnormal 1	Abnormal 2	Abnormal 3
Pitch	Median Pitch (Hz)	222.953	265.220	312.707	320.426
	Mean Pitch (Hz)	212.888	253.116	319.623	320.983
	Standard Deviation (Hz)	59.335	41.830	57.155	25.249
	Minimum Pitch (Hz)	75.385	88.762	77.668	273.255
	Maximum Pitch (Hz)	281.755	278.022	394.755	363.302
Pulses	Number of Pulses	69	135	116	182
	Number of Periods	67	130	112	177
	Mean Period (s)	4.548677E-3	3.915903E-3	3.168996E-3	3.145089E-3
	Standard Deviation of Period (s)	1.858438E-3	0.938877E-3	1.111028E-3	0.404577E-3
Voicing	Fraction of Locally Unvoiced Frames (%)	11.207	11.282	45.667	3.409
	Number of Voice Breaks	1	2	1	0
	Degree of Voice Breaks (%)	5.269	17.195	48.111	0

**Table 4:** The jitter, shimmer, and harmonicity across the conditions of the sound / ħ /.

Acoustic features	Parameter	Normal	Abnormal 1	Abnormal 2	Abnormal 3
Jitter	Jitter (Local) (%)	2.624	1.948	2.370	2.440
	Jitter (Local, Absolute) (s)	119.369E-6	76.274E-6	75.099E-6	76.753E-6
	Jitter (Rap) (%)	0.709	1.048	1.256	1.214
	Jitter (Ppq5) (%)	0.842	1.198	1.495	1.415
	Jitter (Ddp)	2.126	3.143	3.769	3.643
Shimmer	Shimmer (Local) (%)	10.736	9.182	22.078	9.870
	Shimmer (Local, dB)	0.965	1.024	1.926	1.048
	Shimmer (Apq3) (%)	5.111	4.503	10.855	4.618
	Shimmer (Apq5) (%)	6.626	6.542	13.543	7.530
	Shimmer (Apq11) (%)	11.383	7.795	19.579	9.459
	Shimmer (Dda)	15.332	13.508	32.564	13.853
Harmonicity	Mean Autocorrelation	0.847334	0.834286	0.757948	0.834019
	Mean Noise-to-Harmonics Ratio	0.229948	0.261485	0.362134	0.295336
	Mean Harmonics-to-Noise Ratio (dB)	9.381	10.217	5.562	11.735

**Table 5:** The pitch, pulse1, and voicing across the conditions of the sound / ʕ /.

Acoustic features	Parameter	Normal	Abnormal 1	Abnormal 2	Abnormal 3
Pitch	Median Pitch (Hz)	302.353	255.970	312.078	285.573
	Mean Pitch (Hz)	282.764	252.335	316.241	286.715
	Standard Deviation (Hz)	36.212	12.091	46.187	13.408
	Minimum Pitch (Hz)	210.475	206.762	243.718	264.180
	Maximum Pitch (Hz)	342.972	265.264	389.262	317.732
Pulses	Number of Pulses	206	137	127	153
	Number of Periods	202	134	123	150
	Mean Period (s)	3.554860E-3	4.068388E-3	3.222346E-3	3.488968E-3
	Standard Deviation of Period (s)	0.521071E-3	0.600651E-3	0.499375E-3	0.185627E-3
Voicing	Fraction of Locally Unvoiced Frames (%)	7.563	4.520	40.217	2.454
	Number of Voice Breaks	0	0	1	0
	Degree of Voice Breaks (%)	0	0	2.212	0

Table 3 provides an in-depth analysis of the voice parameters for normal and abnormal individuals’ pronunciations. The analysis offers different functions and qualities of certain occurrences, focusing on Arabic pharyngeal sounds. The table focuses on essential parameters related to sound analysis, such as pitch, pulse, and voicing. The Pitch parameters focus on median, mean, standard deviation, minimum, and maximum measurements.

According to Table 3, the trend of increasing the pitch across normal and abnormal is evident, mainly in the abnormal three. The voice in abnormal states is higher in pitch because of the increased tension and stiffness in the vocal folds. The standard deviation decreases in the state of the third abnormal individual, suggesting that the pitch variability tends toward more abnormal individuals. In addition to the higher pitch, the abnormal 3 indicates a restricted range because of vocal fold dysfunction. For the minimum measures, there is a loss in low-pitch control in the case of abnormals. The pitch increases to the maximum peak for abnormal 2, reflecting strain effort in voice production. The number of pulses and periods increases in the state of abnormal 3, which indicates an increase in the vibration of the vocal folds because of compensatory efforts to maintain the production of sounds despite pathological changes. The mean Period from normal to abnormal 3 indicates quick vocal cord vibrations. It indicates more consistent period durations of rigid or less dynamic vocal systems, as shown in the decrease in standard deviation. In the case of voicing, there is a significant increase in abnormal 2, representing a higher proportion of settings without voicing, and that indicates a severe disruption in the closure of the vocal cords. The variation in the voice breaks, where, in some states, there is a significant and frequent interruption in voicing, as shown in Table 2; Abnormal 2 indicates the number of voice breaks. However, abnormal 3 shows no voice breaks, which leads to various speech disorder symptoms. Table 3 highlights the changes in the progression of voice abnormalities, which is crucial for understanding the underlying pathology and leading to clinical assessment and intervention in speech disorders.

Figure 2 displays the pitch, pulse, and voicing parameter variations across different conditions. These graphs collectively illustrate the detailed visual insights represented in each aspect of voice quality alterations across the range of normal and abnormal individuals’ states, highlighting some instances of change in voice characteristics.

There was high variability in the voicing analyses in the fraction of unvoiced frames and the voice breaks. The major disruptions occurred in Abnormal 2, because it recorded very high percentages of local unvoiced frames, and voice breaks were frequent, suggesting serious glottal closure problems. Intriguingly, voice breaks were scarce in Abnormal 3, which points to different manifestations of speech pathology symptoms.

Voice quality parameters showed complex underlying tendencies for jitter, shimmer, and harmonicity changes, as shown in Table 4. Jitter and shimmer, representing pitch and amplitude variability, respectively, reached the highest values in Abnormal 2, implying extreme deviation from normal phonation and perceived voice roughness. In contrast, Abnormal 1 and 3 had values of jitter and shimmer closer to normal, in line with more controlled vocal production. The harmonicity analysis, based on the harmonics-to-noise ratio measurement, revealed problems in the abnormal two-voice quality owing to increased noise levels. On the other hand, though still problematic, Abnormal 3 shows better harmonic structuring. Each individual with a speech disorder exhibits unique deviations from the normal speech profile, emphasizing the need for personalized diagnostic and therapeutic approaches. Figure 3 visualizes how jitter, shimmer, and harmonicity parameters are affected under different conditions and how the pathological cases are altered.

The summarized distributions from Figures 2, 3 point toward an increasing abnormality trend across conditions. Pitch values, pulsing, and voice variability mainly increased or showed marked deviation from normal behavior in abnormal states. Shimmer demonstrated amplitude perturbations in Abnormal 2, while jitter appeared to indicate higher pitch stability in Abnormal 3. Together, these results suggest that there exist minute differences among people with speech disorders, hence giving rise to an individualized approach to the assessment and treatment of speech disorders involving Arabic pharyngeal sounds.

### 5.2 An acoustic analysis of the sound (/ ʕ/, / ع/)

**Figure 4:**
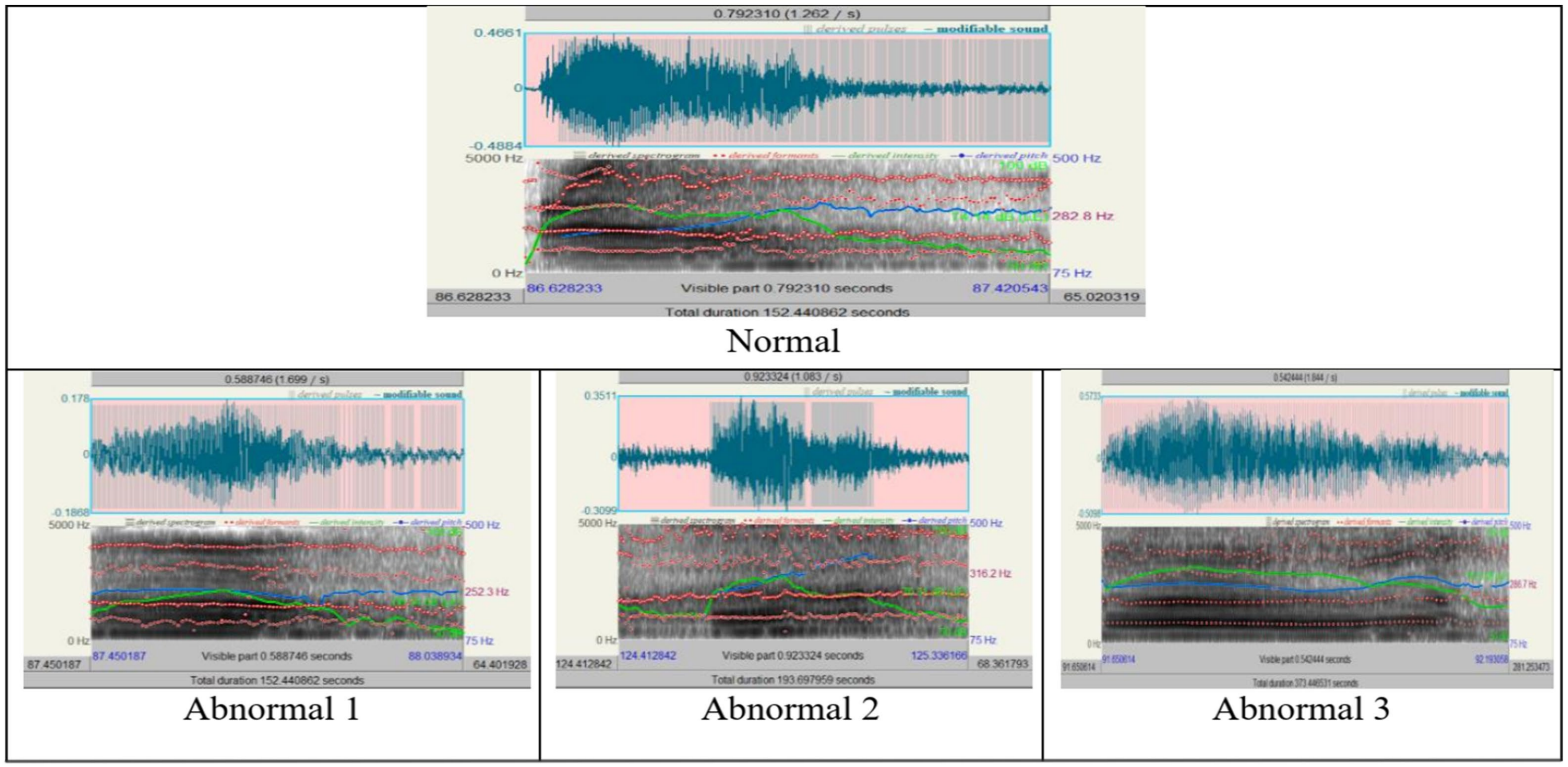
The acoustic analyses of the sound / ʕ /, “/ ع/”.

**Figure 5:**
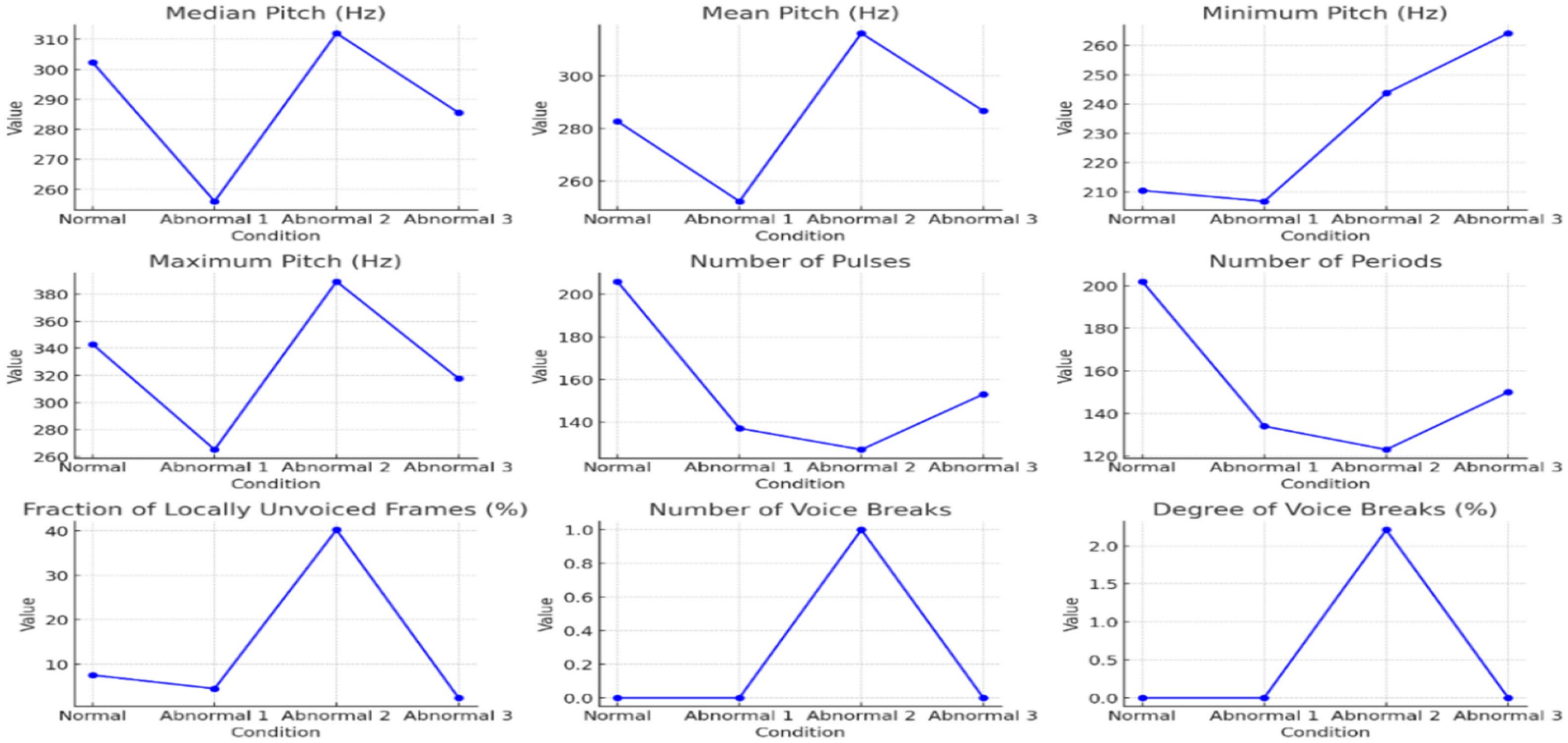
The pitches, pulse, and voicing change in different conditions of the sound / ʕ /.

**Figure 6:**
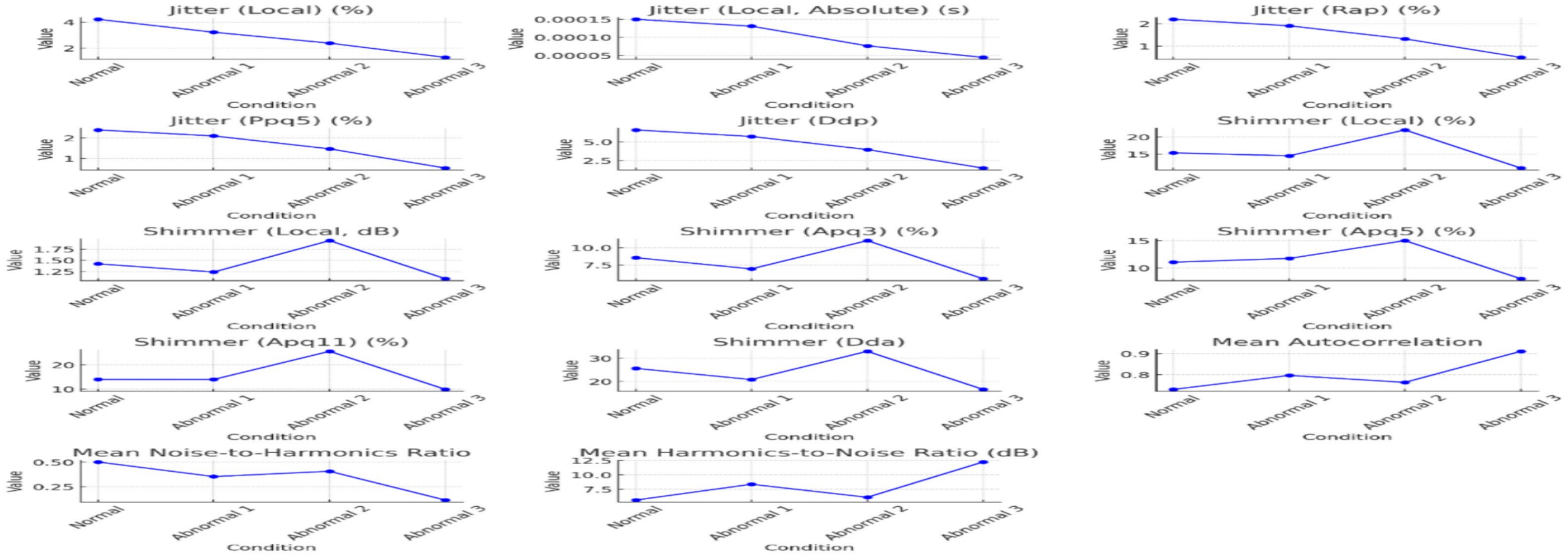
The jitter, shimmer, and harmonicity parameters across the conditions of the sounds of / ʕ /.

The second sound (/ʕ/) acoustic analysis was conducted in normal and abnormal conditions to assess the voice characteristics, such as the regularity of the waveform, pitch stability, and clarity of formant structures (Figures 4–6; Tables 4–6). Figure 4 illustrates the differences among them by analyzing the waveform, spectrogram, and pitch contour. Normal waveforms exhibited regular rhythmic patterns, stable pitch contours with natural variability, and formant structures with clear definition, thus suggesting precise articulatory control. On the other hand, abnormal waveforms showed increased variability of the amplitudes, irregular voicing, disruptions of airflow, and reduced clarity of formant structures, thus suggesting imprecise articulation, hoarseness, and breathiness. The pitch contours were highly unstable, with irregular voicing in Abnormal 2, as in Abnormal 3, though relatively consistent in the control of the waveform and pitch, and still showed some articulatory imprecision.

**Table 6:** The jitter and shimmer values across individuals of articulating the sound / ʕ /.

Acoustic features	Parameter	Normal	Abnormal 1	Abnormal 2	Abnormal 3
Jitter	Jitter (Local) (%)	4.225	3.220	2.378	1.277
	Jitter (Local, Absolute) (s)	150.184E-6	131.011E-6	76.614E-6	44.549E-6
	Jitter (Rap) (%)	2.196	1.907	1.325	0.502
	Jitter (Ppq5) (%)	2.385	2.095	1.469	0.538
	Jitter (Ddp)	6.589	5.720	3.976	1.506
Shimmer	Shimmer (Local) (%)	15.347	14.472	22.023	10.900
	Shimmer (Local, dB)	1.422	1.239	1.940	1.089
	Shimmer (Apq3) (%)	8.553	6.950	10.993	5.540
	Shimmer (Apq5) (%)	11.062	11.743	15.019	8.018
	Shimmer (Apq11) (%)	14.044	14.020	25.580	9.923
	Shimmer (Dda)	25.658	20.849	32.978	16.620
Harmonicity	Mean Autocorrelation	0.731014	0.796932	0.764267	0.910852
	Mean Noise-to-Harmonics Ratio	0.503484	0.354627	0.408413	0.114442
	Mean Harmonics-to-Noise Ratio (dB)	5.652	8.381	6.136	12.228

The analyses of parameters such as pitch, pulse, voicing, jitter, shimmer, and harmonicity have been intertwined in Figures 5, 6, emphasizing the central tendency, variability, and influential outliers between conditions. Pitch and jitter/shimmer variability measurements stressed significant differences between normal and abnormal conditions.

The statistical analysis in Table 4 indicates that the vocal cords of a normal person show greater width and variability compared to an abnormal 1, indicating a more active and expressive speech ability. Abnormal 3 has vowel values closer to the normal range, which means more control than Abnormal 2. Nevertheless, the expressed vowels have their nuances. The number of pulses and periods, as well as their mean and standard deviation, vary from person to person, which means differences in intonation and fluency. It indicates a dramatic wasting of voice, unlike the more vocal hoarseness seen in normal and other abnormal cases. More specifically, these were the parameters that were mainly changed, with pitch (median and mean pitch) showing considerable dispersion across the four conditions, more marked in Abnormal 2, hence, indicating possible destruction of normal vocal control mechanisms.

Jitter parameters decreased frequency perturbation from Normal to Abnormal 3, hence indicating less pitch variability with aggravation of abnormality. Shimmer parameters conversely showed maximal amplitude instability in Abnormal 2, hence indicating maximum problems in speech clarity, which started recuperating in Abnormal 3.

Harmonicity measures, such as mean autocorrelation and harmonics-to-noise ratio, demonstrated that Abnormal 3 had more clarity and consistency of the voice signal compared with the other abnormal conditions, albeit with articulatory difficulties. In particular, Abnormal 2 recorded severe deterioration in voice quality metrics: high levels of shimmer and noise.

Table 6 shows that Jitter and shimmer values across individuals reflect differences in pitch and amplitude stability. Abnormalities 2 and 3 indicate jitter changes and differences in frequency control. Shimmer values, exceptionally high in the abnormal range of 2, show considerable amplitude variability, affecting speech clarity and perceived quality. The harmonic-to-noise ratio and average autocorrelation values provide insight into the nature of the sound. Unusual 3 exhibits high consistency, showing clear voice characteristics despite other speech difficulties, whereas abnormal 2 shows a mixture of characteristics, with some aspects suggesting possible qualitative relevance. This comparative study emphasizes the complex effects of speech on various acoustic difficulties.

The findings above corroborate the statistical data in Tables 4, 6, confirming the vocal differences based on conditions. Abnormal conditions consistently recorded increased heterogeneity for all acoustic parameters, thereby delineating the effect of speech disorders on voice performance. The implications emphasize the need for customized diagnostic and therapeutic procedures for Arabic speakers, which will highlight considerations practical for articulatory accuracy and related focused practices, as stated in previous works (Dodd, 2013; Namasivayam et al., 2020). From this analysis, it can be inferred that speech-language pathologists must employ individualized treatment approaches based on the intricate nature of acoustic and articulatory variations shown by patients with speech disorders, which aligns with a study conducted about speech disorders and uvular sound production in Arabic (Albuhairy et al., 2025).

### 5.3 Neurolinguistic interpretation

The neurolinguistic analysis examines pitch, pulses, and voicing parameters across Normal and Abnormal conditions. The parameters reflect vocal performance, pitch stability, periodicity, and voice continuity and are directly tied to neuromuscular control during speech production.

In normal speech, pitch variability is a hallmark of healthy neural control, allowing speakers to adjust pitch based on prosody and meaning. However, in abnormal conditions, especially in Abnormal 3, we see a significant reduction in pitch variability. This is consistent with disorders affecting the basal ganglia or motor cortex, where neuromotor rigidity can restrict the flexibility of vocal fold movements, leading to higher and more fixed pitch. The reduced mean period and the lower standard deviation of periods in the abnormal conditions reflect impaired rhythmic control, possibly due to damage to the brain’s cerebellum or motor planning areas. These regions are crucial for coordinating the timing of articulatory gestures, and their impairment leads to faster but less controlled speech. The high fraction of unvoiced frames and frequent voice breaks in Abnormal 2 suggest a breakdown in the fine motor control needed to maintain continuous vocal fold vibration, often seen in speech disorders like dysarthria or Parkinson’s disease. The reduction in voice breaks in Abnormal 3, while seemingly an improvement, likely reflects severe neural impairment where speech is reduced to monotonic phonation due to a lack of neural flexibility.

The progression from Normal to Abnormal three conditions reveals increasing neuromotor impairment, with pitch variability, timing, and phonation disruptions. The patterns suggest a loss of flexibility in motor control over the vocal folds, possibly due to damage to the basal ganglia, motor cortex, or cerebellum, all of which play critical roles in modulating speech. As the severity of the speech disorder increases, the neurolinguistic system fails to adjust pitch, timing, and voicing parameters, resulting in more monotonic, rigid, and fragmented speech.

**Figure 7:**
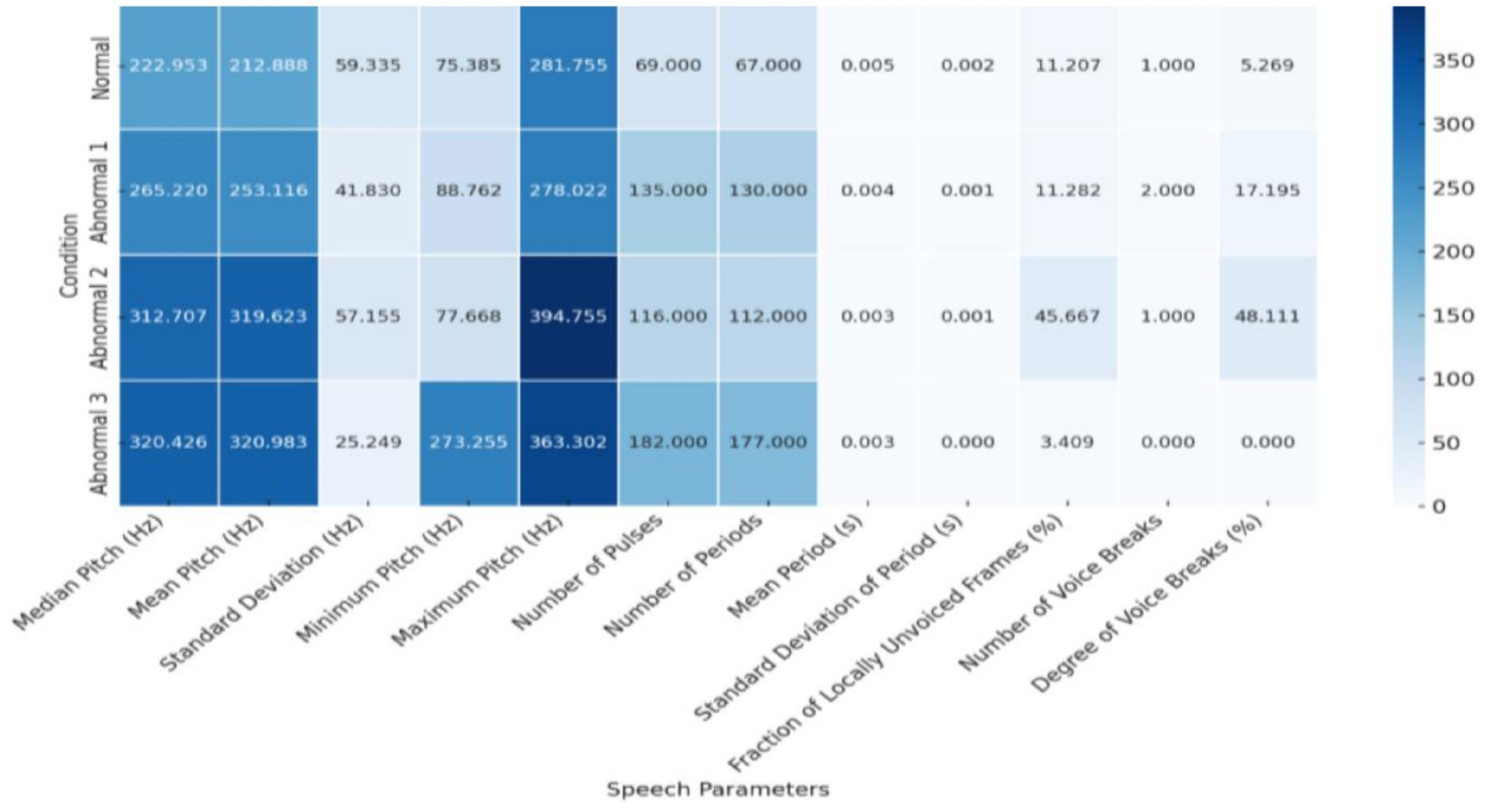
Speech parameters Heatmap across conditions.

Figure 7 highlights the differences across speech parameters (such as pitch, pulses, and voicing) in normal and abnormal conditions. The color intensity reflects the values of each parameter, offering a clear comparison between the conditions.

The neurolinguistic analysis reveals a clear distinction between Normal and Abnormal speech conditions, with the Abnormal two emerging as the most severely affected in terms of pitch variability, rhythmic disruption, and voicing instability. These findings highlight the importance of pitch and rhythm control as critical indicators of neuromuscular impairments in speech disorders. Individuals with more severe abnormalities exhibit significant pitch control and vocal rhythm breakdowns, leading to unstable and inconsistent speech patterns.

And presently, this study of the neuro-acoustic features of the Arabic gutturals would considerably aid therapy and automation in diagnosis by providing concrete parameters of articulatory precision and acoustic consistency, so that speech-language pathologists could design therapeutic exercises aimed at reducing a deficit in one or more areas, such as pitch stability, amplitude control, and phonetic accuracy. The identified acoustic patterns that differentiate normal from abnormal speech states could further be exploited in the automatic diagnosis process, wherein machine learning algorithms could be trained to recognize slight acoustic anomalies related to speech impairment, leading to a quicker and more definite diagnosis and intervention.

## 6 Conclusion

The study’s phonological and neurolinguistic analysis offers a comprehensive understanding of the challenges associated with articulating pharyngeal sounds in Arabic, particularly for individuals with speech disorders. The study highlights the vocal and phonetic characteristics that differentiate normal from disordered speech by identifying fundamental structures and barriers that impact speech clarity and communication. Specifically, it reveals significant acoustic differences, such as variations in pitch, jitter, shimmer, and harmonic-to-noise ratio, between typical speakers and those with speech impairments. These findings underscore the critical role pharyngeal sounds play in maintaining language clarity. Moreover, the neurolinguistic component of the study sheds light on the underlying neurological disruptions that contribute to these speech impairments, emphasizing the need for tailored therapeutic interventions that address the disorder’s acoustic and neural dimensions. The results challenge the effectiveness of a one-size-fits-all approach, advocating instead for individualized treatment plans due to the variability in speech disorders across individuals. The study concludes with a call for a multidisciplinary approach, integrating expertise from linguistics, neurology, technology, medicine, and speech pathology, to develop comprehensive solutions that improve diagnosis, intervention, and, ultimately, the quality of life for individuals with speech disorders.

## Data Availability

The raw data supporting the conclusions of this article will be made available by the authors, without undue reservation.
